# Exendin-4 improves neurodevelopmental outcome after neonatal germinal matrix hemorrhage

**DOI:** 10.1038/s41419-026-09189-9

**Published:** 2026-08-19

**Authors:** Andrea Jonsdotter, Anna-Lena Leverin, Pernilla Svedin, Michelle Lindström, Kerstin Ebefors, Ylva Carlsson, Henrik Hagberg, Eridan Rocha-Ferreira

**Affiliations:** 1 Department of Obstetrics and Gynecology, Region Västra Götaland, Sahlgrenska University Hospital, Gothenburg, Sweden; 2 Centre of Perinatal Medicine and Health, Department of Obstetrics and Gynecology, Institute of Clinical Sciences, Sahlgrenska Academy, University of Gothenburg, Gothenburg, Sweden; 3 Centre of Perinatal Medicine and Health, Department of Physiology, Institute of Neuroscience and Physiology, Sahlgrenska Academy, University of Gothenburg, Gothenburg, Sweden; 4 Department of Physiology, Institute of Neuroscience and Physiology, Sahlgrenska Academy, University of Gothenburg, Gothenburg, Sweden

**Keywords:** Cell death in the nervous system, Disease model

## Abstract

Germinal matrix hemorrhage (GMH) is a common complication in premature infants and is associated with a high risk of neurodevelopmental impairment and mortality. Currently, there are no specific neuroprotective treatments available. Exendin-4 is a drug used for the treatment of type 2 diabetes mellitus, and it has shown neuroprotective effects in several neurological disorders including Alzheimer’s and Parkinson’s disease. In this study, we used the preterm postnatal day 5 rat model of GMH to evaluate whether exendin-4 exerts neuroprotective effects in this setting. Our results show that in the acute phase, exendin-4 reduced microglial activation, caspase-3 activation, p53 expression, AIF-associated cell death, MMP-9 expression, and neutrophil infiltration into the hemorrhage site. Exendin-4 treatment improved neurodevelopmental outcomes in both negative geotaxis and eye-opening latency when compared to saline-treated GMH controls, and conferred gray and white matter protection as early as 48 h after injury, with persistent neuroprotection observed at 5, 11, and 35 days after GMH. Exendin-4-treated animals also showed significant recovery of motor function in the rotarod test. In summary, this study demonstrates that exendin-4 reduces brain injury in a rat model of GMH in both the short and long term and is associated with improved neurological outcome.

## Introduction

Annually, it is estimated that 13.4 million babies are born preterm. One of the main complications arising in very preterm infants is germinal matrix hemorrhage (GMH) [1]. GMH can be divided into four grades based on the Papile classification, with grades III and IV having the worst outcome, associated with a high-rate of mortality, and up to 80% of survivors suffer from cerebral palsy and/or cognitive deficits [2, 3].

In the developing brain, the germinal matrix is a major source of future neurons and glial cells and is a highly vascularized area, which plays a central role in neurodevelopment [4]. Unfortunately, this also makes the germinal matrix selectively susceptible to hemorrhage. Following the initial bleeding, GMH causes blood clots and periventricular/intraventricular hemorrhage (PVH/IVH), leading to compression of brain tissue including the terminal vein and its branches, resulting in disturbances of cerebral blood flow, oxygen delivery and subsequent periventricular hemorrhagic infarction [1]. PVH/IVH further impairs cerebrospinal fluid (CSF) circulation and drainage [5]. The presence of blood products and clot lysis leads to excess release of thrombin, hemoglobin and free iron with subsequent free radical production within the first 72 h of life [6–8]. After resolution of the hematoma, CSF accumulation may lead to hydrocephalus and further tissue compression, resulting in additional cell loss [9, 10]. Once blood flow and tissue pressure were normalized, there is a risk of reperfusion injury associated with continued release of free radicals, sustained oxidative stress, prolonged neuroinflammatory response and further neural cell death. Together, these pathophysiological factors can result in both gray and white matter injury and lead to poor neurodevelopmental outcomes [11, 12].

There is no treatment for GMH once it has occurred. Current management therefore focuses primarily on preventive strategies, including attempts to delay preterm birth and antenatal administration of corticosteroids and magnesium sulfate [13, 14]. Following birth, delayed cord clamping has shown some promise [15]. In cases where hydrocephalus develops, placement of a ventricular shunt may be required [11]. However, in most GMH cases, only supportive care can be provided [16].

Exendin-4 is an agonist of the glucagon-like peptide 1 receptor (GLP-1R) and is used clinically for the treatment of type 2 diabetes mellitus through its ability to regulate blood glucose via stimulation of insulin secretion [17]. Subsequent studies have shown that exendin-4 readily crosses the blood–brain barrier [18] and exerts neuroprotective effects in adult conditions including Alzheimer’s disease [19], Parkinson’s disease [20], stroke [21, 22] and traumatic brain injury [23, 24]. We have previously shown that GLP-1R is expressed in the brain of neonates. We demonstrated that exendin-4 (0.5 μg/g) administered either alone or in combination with therapeutic hypothermia prevented newborn brain injury in the mouse model of hypoxia-ischemia [25, 26], and exendin-4 as a monotherapy markedly reduced brain injury in the rabbit HI model [27]. Based on these findings, the aim of the present study was to evaluate the potentially neuroprotective effect of exendin-4 in a preterm rat model of GMH.

## Methods

### Experimental animals

The present animal study was reviewed and approved by the Swedish Board of Agriculture and approved by the Gothenburg Animal Ethics Committee (825-2017, 3033-2020 and 4031/22). Wistar rats were bred in-house at the Laboratory for Experimental Biomedicine of Gothenburg University. All animals were maintained under normal housing conditions, with a 12 h light/dark cycle and free access to water and food. Animals of both sexes were used for all experiments. All experiments were performed in accordance with the ARRIVE guidelines and relevant institutional regulations. No formal a priori sample size calculation was performed. Group sizes were determined prior to initiation of the experiments based on previous experience with the P5 GMH model and our prior studies using exendin-4, and are consistent with those used in similar preclinical studies, with the aim of detecting biologically relevant effects while minimizing animal use.

### Germinal matrix hemorrhage surgery

At postnatal day (P) 5, Wistar rats were anesthetized with isoflurane (5% induction, 2% maintenance) and GMH was induced via freehand injection of 0.3 U collagenase type VII in 2 µl (Sigma Aldrich, Saint Louis, Missouri, USA; cat# C2399) using a 27 G needle connected to an infusion pump (flow 1 µl/min for 2 min). The needle position was modified from that described by Jinnai et al. to 3 mm lateral of the midline and 0.4 mm rostral of bregma, with the needle angled medially and inserted to a depth of 3.5 mm aiming for the germinal matrix/subventricular zone area [28]. To avoid backflow, the needle remained in place for 1 min extra. The animals were placed on a heating pad (35 °C) and returned to their dams after anesthesia recovery. All animals recovered from this procedure.

### Exendin-4 administration

Exendin-4 (Enzo, Farmingdale, NY, USA; ENZ-PRT111-0001) was administered intraperitoneally (0.5 μg/g body weight) as a four-dose regimen given 12 h apart and starting within 10 min after GMH (GMH + EX4). Saline-treated animals were used as GMH controls (GMH + SAL). Naive animals were included as baseline controls. Prior to group allocation, animals were weighed and sexed, and treatment groups were composed to ensure comparable distributions of body weight and sex across groups, including naïve controls. Animals were then allocated to GMH + SAL and GMH + EX4 groups in a randomized and balanced manner within each litter. Body weight was measured at each injection time-point as well as daily afterwards for all groups.

### Exendin-4 assay

Blood and brain samples were collected at P5 from either naive animals or at 0.5, 2, 6 and 12 h following a single dose of exendin-4. After saline perfusion, samples were collected using EDTA as an anticoagulant and plasma was separated after 10 min centrifugation at 2000 × *g*. The brain tissue was homogenized in PBS and centrifuged for 15 min at 1000 × *g* at 4 °C. The plasma samples and tissue supernatants were analyzed in the Rat Exendin 4 ELISA assay (MyBioSource, San Diego, CA, USA; cat.no. MBS031942) according to the manufacturer protocol.

### Temperature measurements

Core temperature was measured at 0, 12, 24, 36 and 48 h after GMH using a rectal probe (T21, 0.41 mm diameter; Physitemp Instruments, Clifton, NJ, USA).

### Glucose and ketone readouts

Blood glucose and ketone levels (mmol/L) were measured at 0.5, 2, 6 and 12 h using Wellion GALILEO Glucose (WELL10-15) and Ketone (WELL10-10KET) test strips in combination with Wellion GALILEO GLU/KET meter (Wellion, Marz, Austria), according to manufacturer’s instructions.

### Insulin assay

Blood was collected via decapitation at 0.5, 2, 6 and 12 h into Eppendorf tubes and left at room temperature for 30 min before centrifugation at 2000 × *g* for 5 min. Serum was collected and immediately frozen in dry ice. Serum insulin was analyzed by a Rat insulin ELISA kit (ERINS, Invitrogen, Waltham, MA, USA) according to the manufacturer protocol.

### Cytokine assay

Animals were perfused with saline and brain samples were collected at 0.5, 2, 6 and 12 h after GMH and snap-frozen on dry ice. The samples were homogenized in PBS containing 1% Triton X-100, 5 mM EDTA and 1% PIC (Protease Inhibitor cocktail, P8340, Merck, St. Louis, MO, USA). The samples were centrifuged for 15 min at 10,000 × *g* at 4 °C and the supernatants, with a protein concentration of 1 mg/mL, were analyzed in a 5-plex cytokine immunoassay for IL-1β, CXCL1, CCL2, IL-6 and TNFα (Bioplex, Bio-Rad, Hercules, CA, USA).

### Motor function tests

Following GMH-induction a cohort of animals underwent developmental behavioral tests: *Negative geotaxis* test was performed until P10 to quantify the amount of time required for a pup to rotate 180° upwards after being placed head down on a 20° downward slope; *Eye opening* latency was noted until P16 for each eye (Model ppr).

Long-term functional recovery was assessed in a separate cohort of animals: *Rotarod* test was used at P20 and P40 to evaluate motor coordination and balance. The apparatus (Panlab, Harvard apparatus, Holliston, MA, USA) consisted of a horizontal rod (25 cm D) with interlane distances of 5 cm (P20) or 7.5 cm (P40). The rats were placed onto the rod with initial constant speed of 4 rpm. The rotation was accelerated up to 40 rpm over a period of 300 s, with fall latency recorded. Each animal underwent three trials, with an interval of 15–20 min per trial. The apparatus was cleaned after every use with 70% ethanol. The *cylinder rearing* test was used to assess potential motor asymmetry. Animals were placed in a transparent glass cylinder at P21 (14 cm D and 21 cm H) and P41 (17 cm D and 28 cm H) and video recorded for 5 min to quantify forepaw preference during full rearing and lateral exploration [29]. A mirror (30 × 30 cm) was placed at an angle below the cylinder for clear unrestricted observation. The forelimb preference was assessed using the following calculation: (unimpaired-impaired)/(unimpaired + impaired + both) × 100 [30]. Animals that reared less than 10 times within 5 min were excluded from the study [31]. All assessments were performed by a researcher blinded to group allocation.

### Immunohistochemistry

Brains were dissected out at P5, P6, P7, P10, P16 and P41 and immersion fixed in Histofix (Histolab, Gothenburg, Sweden). After, brains were dehydrated and embedded in paraffin and cut with a microtome into 7 μm thick sections, with striatum and hippocampus levels collected for histological assessments. Brain sections underwent deparaffinization in xylene followed by rehydration in graded ethanol. All sections were boiled in 0.01 M citric acid buffer (pH 6.0) for antigen recovery and blocked for both endogenous peroxidase (3% H_2_O_2_) and non-specific antigen-binding (5% serum). Consecutive sections were incubated overnight at 4 °C with the following primary antibodies: Mouse anti-microtubule-associated protein-2 (MAP-2; cat# M4403; 1:1 000, Sigma-Aldrich), mouse anti-myelin basic protein (MBP; SMI-94, cat# 836504; 1:1 000, BioLegend, SanDiego, CA, USA), rabbit anti-ionized calcium-binding adapter molecule 1 (IBA-1; cat# 019-19741; 1:2 000, FUJIFILM Wako Chemicals, Osaka, Japan,), rabbit anti-active caspase-3 (cat# 559565; 1:100, Pharmingen, Franklin Lakes, NJ, USA,), anti-p53 (cat# 2524; 1:500, Cell Signaling Technology, Danvers, MA, USA) counterstained with eosin, anti-matrix metalloproteinase-9 (MMP-9; cat# AB228402; 1:250, Abcam, Cambridge, UK) and rabbit anti-myeloperoxidase (MPO; cat#AB9535; 1:100 Abcam). Sections were then incubated with appropriate secondary antibody, followed by ABC incubation (ABC Elite, Vector labs, Newark, CA, USA). Immunoreactivity was visualized using 0.5 mg/ml 3.3-diaminobenzidine enhanced with nickel sulfate. All sections were dehydrated in increasing concentrations of ethanol, followed by xylene, and then covered using Pertex (Histolab).

### Immunofluorescence

Brain sections were deparaffinized, rehydrated, and boiled in citric acid buffer (0.01 M, pH 6.0, 10 min). Laminin and Claudin 5 sections were treated with Proteinase K (10 µg/ml in PBS, Roche, Basel, Switzerland) for 10 min and autofluorescence was blocked before staining with TrueBlack® (Biotium, Fremont, CA, USA). After blocking in 5% serum for 1 h at room temperature, the sections were incubated for 2 h at room temperature with the following antibodies: GLP-1R (cat# NBP-1-97308; 1:100, Novus Biologicals, Centennial, CO, USA), neuronal specific nuclear protein (NeuN; cat# MAB377; 1:250, Merck Millipore Burlington, MA, USA), glial fibrillary acidic protein (GFAP; cat# G3893; 1:500, Sigma Aldrich), ionized calcium-binding adapter molecule 1 (IBA-1; cat# ab5076; 1:250, Abcam), oligodendrocyte transcription factor 2 (Olig2; cat# MABN50; 1:500, Merck Millipore), apoptosis inducible factor (AIF; cat# ab32516; 1:100, Abcam), laminin (cat# NB300-144; 1:200, Novus Biologicals) or claudin 5 (Cat# 35-2500, 1:50 ThermoFischer, Waltham, MA, USA) in 5% serum and 0.1% Triton in PBS. The following AlexaFluor secondary antibodies (ThermoFischer) were used: donkey-anti-goat 488 (A11055), donkey-anti-rabbit 594 (A21207), goat-anti-mouse 488 (A11001), goat-anti-rabbit 594 (A11012), goat-anti-mouse 594 (A11032) and goat-anti-Rabbit 488 (A11008) in 1:500 dilutions in PBS and incubated for 1 h at room temperature. The sections were mounted with a coverslip using ProLong™ Gold + DAPI (P36935, ThermoFisher). Colocalization was examined and photographed using a Zeiss LSM 800 confocal microscope (Zeiss, Oberkochen, Germany). AIF quantification was performed using a Zeiss Imager Z2 (Zeiss). All images were processed using ZEN blue, version 3.4 (Zeiss).

### Data analysis

All assessments were performed by a researcher blinded to group allocation. *Tissue loss* was measured for gray (MAP-2) and white matter (MBP) brain tissue at the striatum and anterior hippocampus levels. Total staining positive area was delineated in each hemisphere and measured using ImageJ (version 1.54 h, NIH, Bethesda, MD, USA). The percentage of tissue/ subcortical white matter loss was obtained by subtracting the ipsilateral from the contralateral area [25]. *Microglial activation* was assessed using a semi-quantitative score of IBA-1+ cells consisting of: 0-no activation, ramified microglia; 1-ramified microglia with focal activation; 2-mild diffuse activation with predominant ramified microglia; 3-moderate widespread activation with microglia showing retracted processes and swollen cell bodies; 4-widespread amoeboid microglia [32]. *Neutrophil* and *Caspase-3* measurements were performed at ×40 as quantification of the total number of positive cells per section/region. The total number of *p53, MMP-9* and *Cytoplasmic/Nuclear AIF* cells was quantified at ×40 in the penumbra region surrounding the bleeding site. *Ipsilateral ventricle dilation* was quantified at the striatum level using ImageJ (NIH) and values expressed as mm^2^. Hematoma size was determined by identifying the first and last coronal sections containing visible hemorrhage. *Hematoma area* was measured in these sections and in representative sections between them using ImageJ (NIH). *Hematoma volume* was estimated by integrating the measured areas across the rostrocaudal extent of the lesion.

### Statistics

GraphPad Prism 10.0 (GraphPad Software, Boston, MA, USA) was used to perform all statistical analyses. The data was first tested for Gaussian distribution using the D’Agostino & Pearson test. Mann–Whitney, Kruskal–Wallis with Dunn’s post hoc test or ANOVA with Tukey’s multiple comparisons test were used when appropriate to determine statistical significance. The statistical test used in each experiment is stated in the corresponding figure legend. A *P* value < 0.05 was considered statistically significant. Data are presented as individual animals or as mean ±SEM.

## Results

### GLP-1R expression and brain uptake of exendin-4

**Fig. 1:**
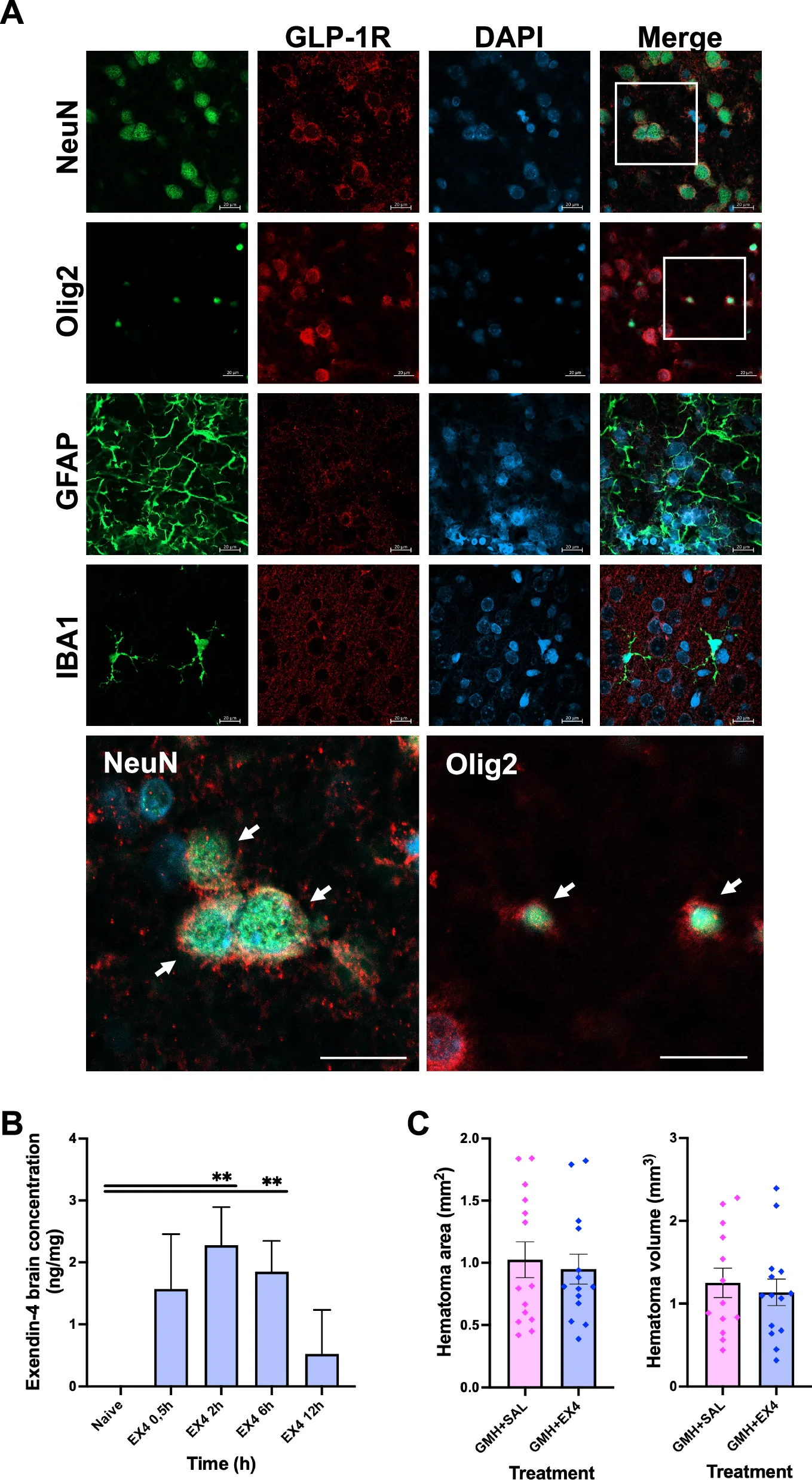
GLP-1R expression and exendin-4 effect in the GMH model. **A** Confocal microscopy imaging performed in naive P5 rats revealed GLP-1R expression was predominantly observed in neurons (NeuN), but it is also present in some oligodendrocytes (Olig2). GLP-1R was not detected in astrocytes (GFAP) and microglia (IBA-1) cells. White arrows indicate colocalization. Scale bar = 20 µm and = 40 µm (inserts). **B** Exendin-4 detection assay confirmed brain penetrance of peripherally injected exendin-4 within 0.5 h post-injection (*n* = 10 per time-point/group). **C** Measurement of both hematoma area and volume showed that exendin-4 did not affect the extent of bleeding 6 h post-GMH (*n* = 14/group). Data presented as individual animals or mean ± SEM and analyzed using unpaired *t*-test. *P < 0.05 and **P < 0.01.

Immunofluorescence colocalization between GLP-1R and neural cell markers demonstrated that GLP-1R is present in the brain of neonatal P5 rats, more specifically in neurons (NeuN), and to a smaller extent, in oligodendrocytes (Olig2). There was no GLP-1R colocalization observed with astroglia (GFAP) or microglia (IBA-1) (Fig. 1A).

Intraperitoneal injection of exendin-4 in P5 naive animals resulted in rapid brain uptake of exendin-4, which reached significant levels at 2 h (*P* = 0.0089) and 6 h (*P* = 0.0173) when compared to naive controls. Brain exendin-4 levels were largely cleared by 12 h (Fig. 1B).

To determine whether exendin-4 affected the hemorrhagic injury, the extent of GMH-mediated bleeding was evaluated at 6 h post-GMH, a time-point when the collagenase-induced hemorrhage site is largest [33]. No difference in hematoma area or volume was observed between GMH + SAL and GMH + EX4 animals (Fig. 1C).

### Physiological and biochemical measurements

**Fig. 2:**
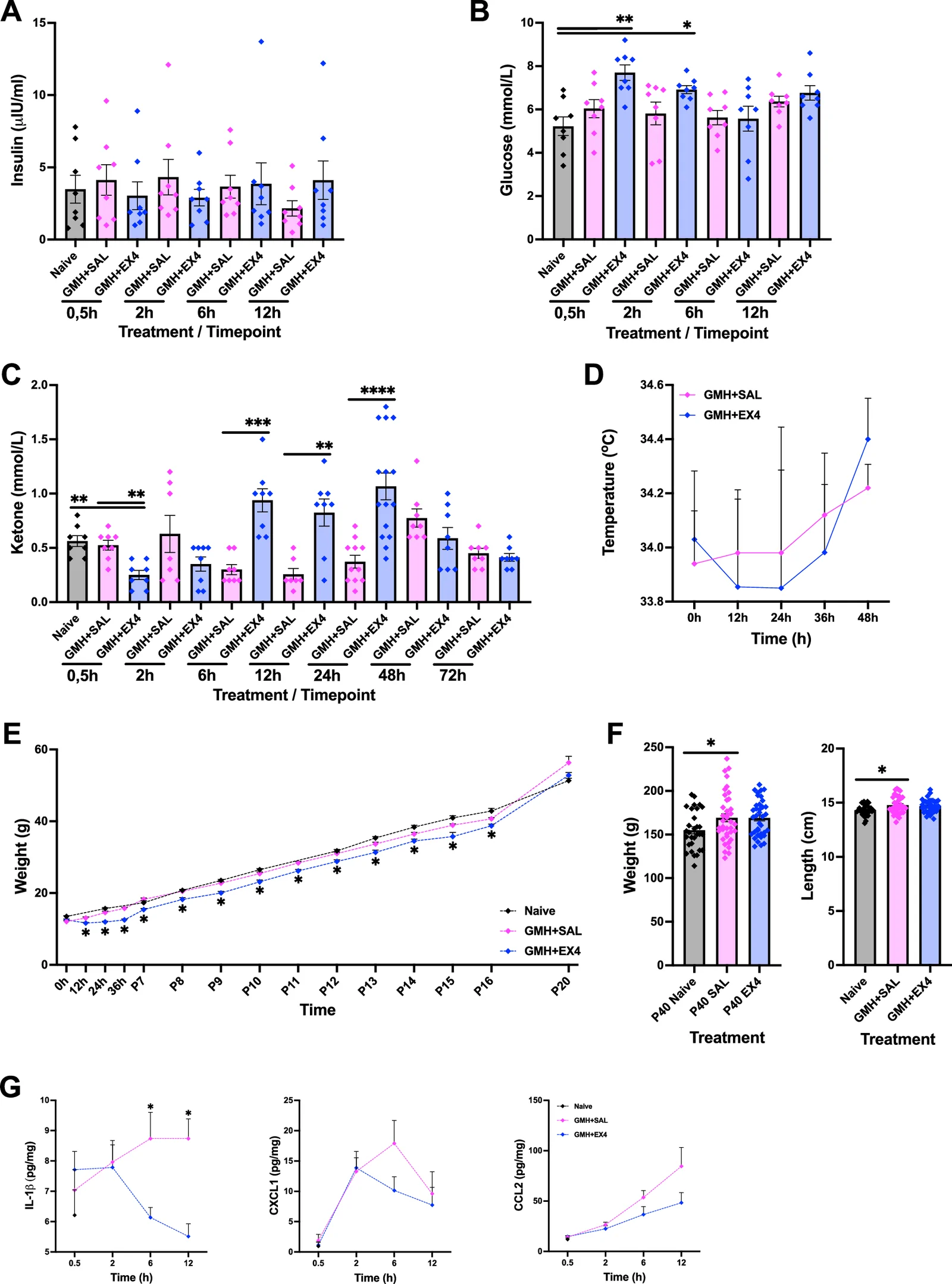
Physiological measurements following a single dose of exendin-4. **A** Insulin and **B** glucose blood levels were measured 0.5, 2, 6 and at 12 h after saline or a single exendin-4 administration (*n* = 8/group/time-point). Naive animals (*n* = 8) were used as baseline controls. **C** Ketone bodies in blood were also measured at 0.5, 2, 6 and 12 h (single injection), as well as at 24 h (2 injections), 48 h and 72 h (4 injections) (*n* = 8/group/time-point). **D** Body temperature was measured at each exendin-4 injection time-point and at 48 h (12 h after last injection). **E** Body weight was recorded at each exendin-4 injection time-point followed by once daily until P16. Body weight was again recorded at P20 and **F** P40, the latter together with body length. **G** IL-1β, CXCL1 and CCL2 levels in the brain were measured at 0.5, 2, 6 and 12 h after GMH combined with either saline or exendin-4 treatment, with naive animals used as baseline controls (*n* = 8/group/time-point). Data presented as individual animals or mean ± SEM and analyzed using Kruskal–Wallis with Dunn’s post hoc test. *P < 0.05, **P < 0.01, ***P < 0.001 and ****P < 0.0001.

Following GMH, a single dose of exendin-4 did not affect blood insulin levels (Fig. 2A). However, the same animals showed a significant increase in glucose at 0.5 h (*P* = 0.0023) and 2 h (*P* = 0.0172) when compared to naive controls (Fig. 2B).

Blood ketone body levels in the GMH + EX4 group were initially lower (0.5 h) when compared to both naive (*P* = 0.0032) and GMH + SAL (*P* = 0.0094) animals. By 6 h after exendin-4 administration there was a significant increase in ketone bodies (P = 0.0001) which remained high at 12 h (*P* = 0.0026) and at 24 h (*P* < 0.0001) when compared to GMH + SAL, with normalization by 48 h after GMH (Fig. 2C). Temperature measurements recorded at each exendin-4 injection time-point showed no difference between the GMH + SAL and GMH + EX4 groups (Fig. 2D).

Exendin-4 injection resulted in significant reduction in weight gain throughout treatment starting at 12 h post-GMH (12 h, *P* = 0.0004; 24 h, *p* < 0.0001; 36 h, *p* < 0.0001). At 48 h after GMH, exendin-4 animals resumed weight gain in parallel to both naive and GMH + SAL controls, however, GMH + EX4 animals still had lower weight until P16. By P20, i.e., 15 d after GMH, all groups had similar weights (Fig. 2E). Body weight measurements at P40 showed that GMH + EX4 animals had both normalized body weight and body length when compared to naive controls, whereas GMH + SAL animals were both heavier and larger than naive littermates (Fig. 2F).

Measurements of cytokines in brain homogenates demonstrated that a single exendin-4 dose inhibited the increase in IL-1β levels compared to GMH + SAL controls at 6 h (*P* = 0.0474) and 12 h (*P* = 0.0064) post-GMH. CXCL1 increased significantly at 2 h in both GMH + SAL (*P* = 0.0041) and GMH + EX4 (*P* = 0.0061) groups. However, by 6 h CXCL1 levels started to normalize in GMH + EX4 animals, whereas it remained high in GMH + SAL when compared to naive controls (*P* = 0.0008). Overall, GMH + EX4 animals had lower CCL2 levels compared to GMH + SAL animals (Fig. 2G). The levels of IL-6 and TNFα were below the limit of detection in both the GMH + SAL and GMH + EX4 groups across the time points analyzed.

### Acute effects during exendin-4 treatment

**Fig. 3:**
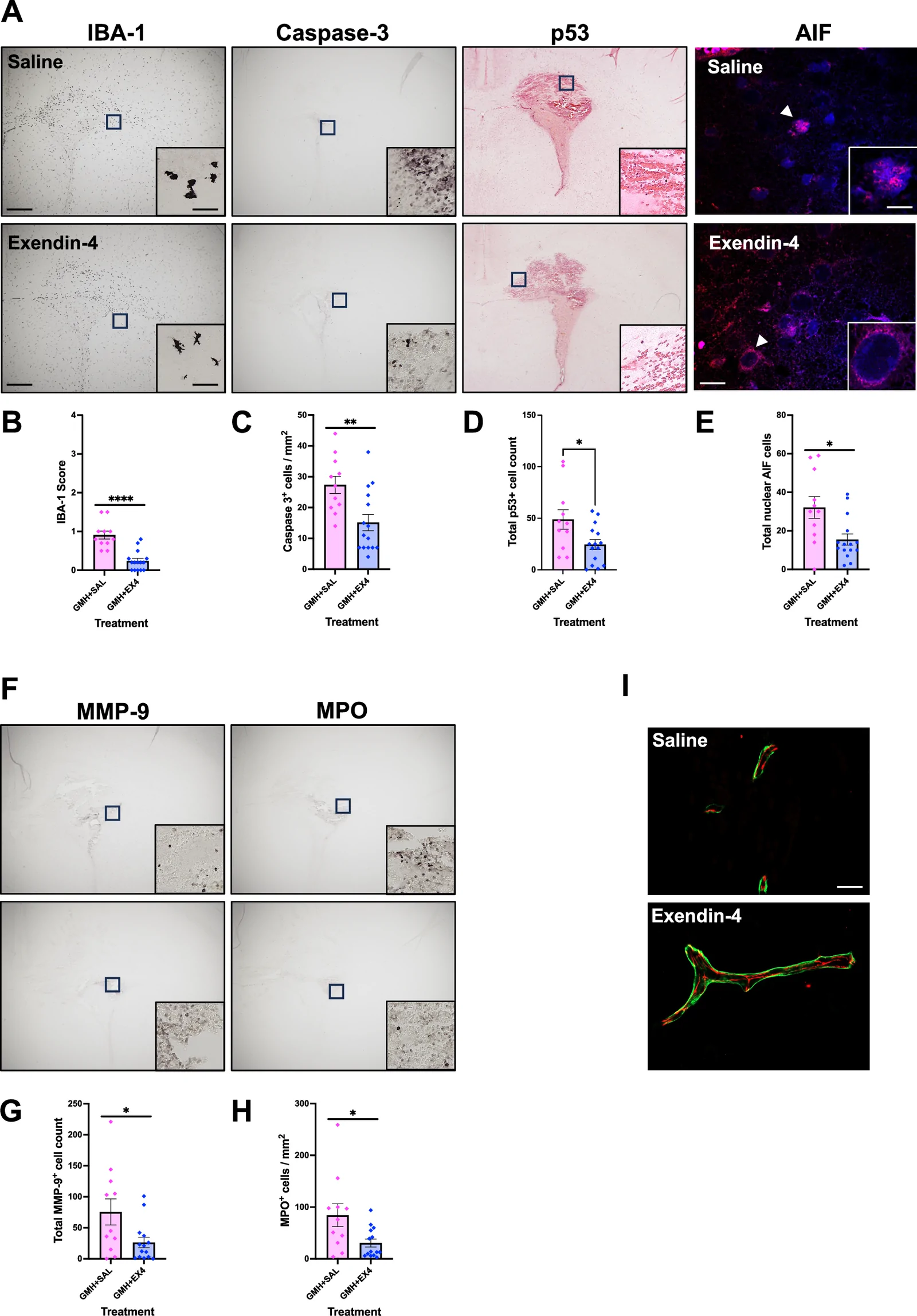
Exendin-4 reduces several pathological processes within 24 h post-GMH. **A** Representative micrographs of microglia (IBA-1), caspase-3, p53 and AIF immunohistochemistry. Corresponding quantitative measurements, including (**B**) IBA-1 scoring, **C** caspase-3 and **D** p53 cell quantification, and **E** AIF nuclear translocation. **F** MMP-9 and neutrophil (MPO) immunohistochemistry, with corresponding (**G**) MMP-9 and **H** MPO quantification in both GMH + SAL (*n* = 11) and GMH + EX4 (*n* = 15) animals. **I** Representative immunofluorescence showing claudin-5 (red) and laminin (green) expression in the peri-hemorrhagic region. Data presented as individual animals or mean ± SEM and analyzed using the Mann–Whitney test. *P < 0.05, **P < 0.01 and ****P < 0.0001. Scale bar = 0.5 mm and 500 μm (insert) or 20 μm (**I**).

At 24 h after GMH, animals that received 2 doses of exendin-4 (12 h apart) showed a significant decrease in several injury-associated markers. There was a substantial reduction in microglial activation (*P* < 0.0001; Fig. 3A, B), cleaved caspase-3 (*P* = 0.006; Fig. 3A, C) and p53 (*P* = 0.0376; Fig. 3A, D) when compared to GMH + SAL controls. Additionally, exendin-4 treatment resulted in 56% reduction in nuclear translocation of AIF (from 35 ± 5 to 15 ± 3, *P* = 0.0014; Fig. 3A, E). The MMP-9 total cell count was significantly lower in GMH + EX4 than GMH + SAL animals (*P* = 0.0457; Fig. 3F, G). Exendin-4 also reduced the infiltration of neutrophils (MPO+ cells) compared to the saline group (*P* = 0.0275; Fig. 3F, H). Qualitative immunofluorescence colocalization of laminin (green) and claudin-5 (red) indicated preservation of vasculature in the peri-hemorrhagic site of GMH + EX4 animals (Fig. 3I).

### Exendin-4 reduces acute injury at 48 h after GMH

**Fig. 4:**
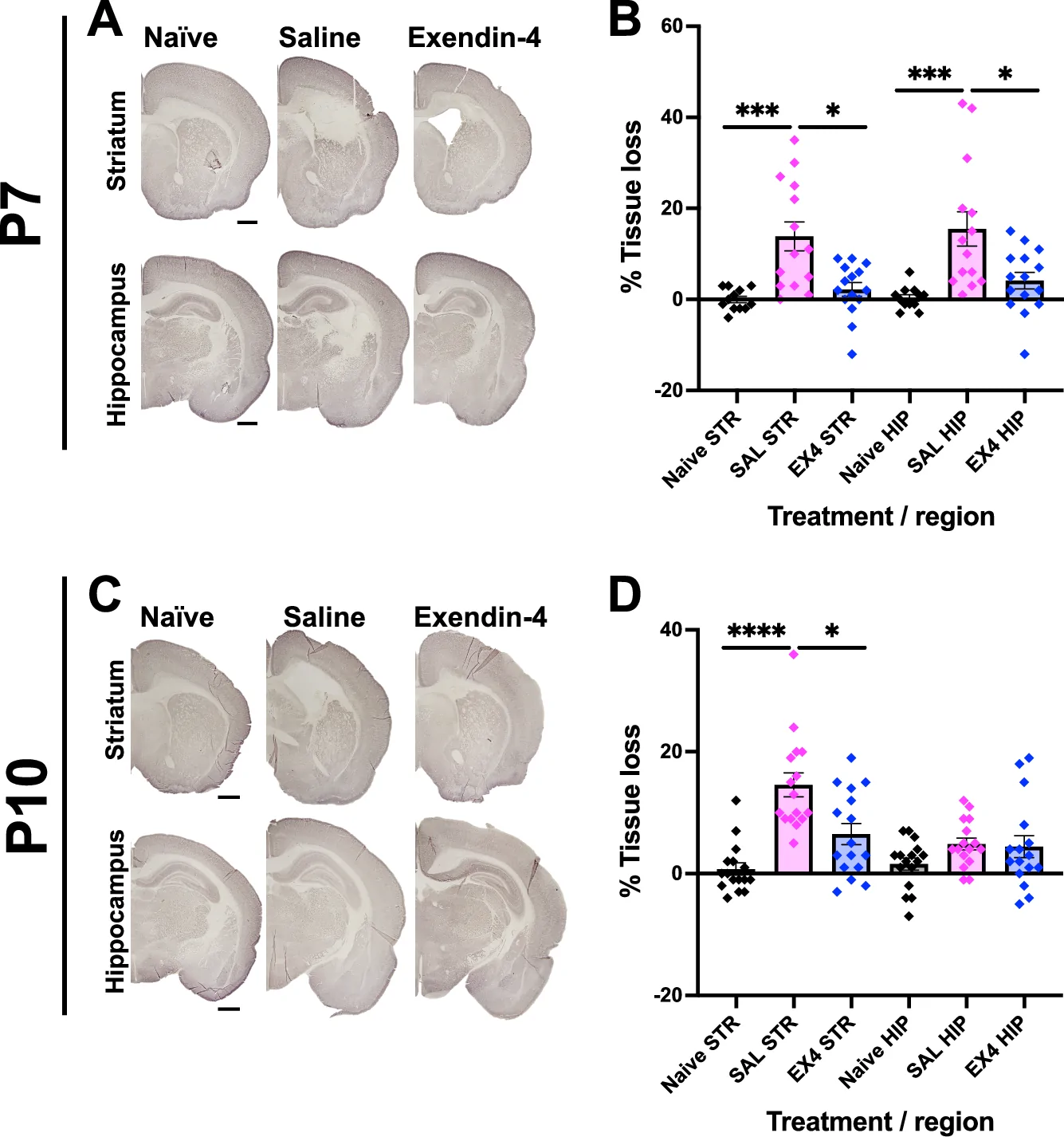
Exendin-4 administered as a four-dose regimen results in early neuroprotection. **A** Representative micrographs of MAP-2-stained striatum and hippocampus brain regions of naive, GMH + SAL and GMH + EX4 animals at P7, i.e. 48 h after GMH induction, and **B** corresponding tissue loss percentage measurements in the three different treatment groups: Naive (*n* = 12), GMH + SAL (*n* = 14) and GMH + EX4 (*n* = 15). **C** P16 (11 d post-GMH) representative micrographs of MAP-2-stained striatum and hippocampus brain regions of naive, GMH + SAL and GMH + EX4 animals and **D** equivalent tissue loss percentage measurements in the three different treatment groups (*n* = 16/group). Data presented as individual animals or mean ± SEM and analyzed using Kruskal–Wallis with Dunn’s post hoc test. *P < 0.05, **P < 0.01, ***P < 0.001 and ****P < 0.0001. Scale bar = 1 mm.

Evaluation of gray matter brain injury based on MAP-2 immunohistochemistry demonstrated that exendin-4 reduced tissue loss in the hippocampus (*P* = 0.0368), while showing a trend toward reduction in the striatum (*P* = 0.0529) brain levels at 48 h-post-GMH (Fig. 4A, B).

Repeated evaluation at P10 showed continued significant protection in the striatum (*P* = 0.045). There was no detectable brain injury at the hippocampal level in GMH + SAL animals (Fig. 4C, D).

### Exendin-4 sustains long-term neuroprotection

**Fig. 5:**
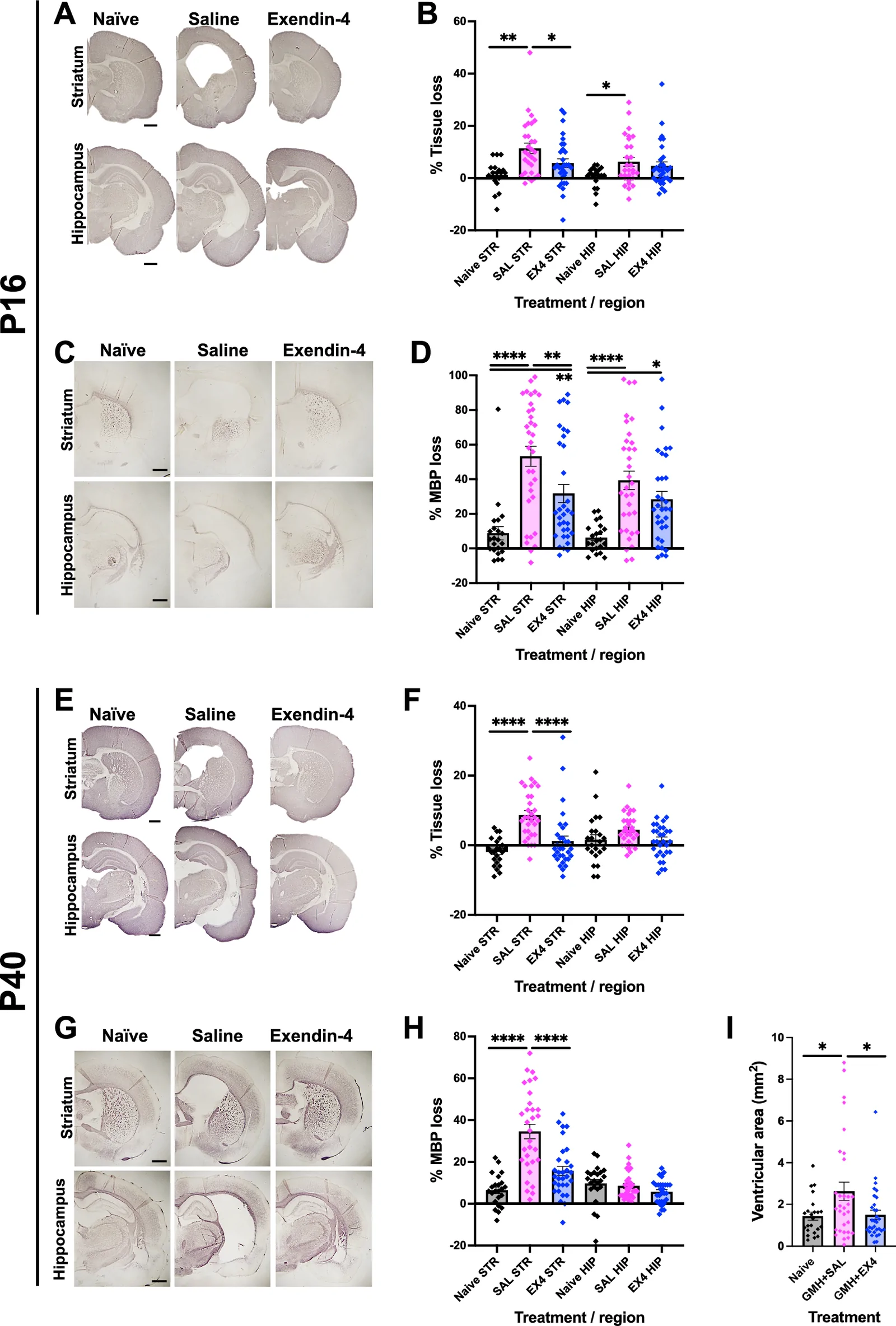
Exendin-4 treatment results in long-term anatomical neuroprotection. **A** Representative micrographs of MAP-2-stained striatum and hippocampus brain regions of naive, GMH + SAL and GMH + EX4 animals and **B** respective MAP-2 tissue loss percentage measurements at P16, i.e. 11 d after GMH. **C** Representative micrographs of MBP-stained white matter in the striatum and hippocampus brain levels of the same animals and **D** corresponding subcortical white matter loss measurements in the three different treatment groups. Sample sizes were: Naive (*n* = 23), GMH + SAL (*n* = 31), GMH + EX4 (*n* = 31). **E** P40 (25 d after GMH) representative micrographs of MAP-2-stained striatum and hippocampus brain regions of naive, GMH + SAL and GMH + EX4 animals and **F** matching MAP-2 tissue loss percentage measurements in the three different treatment groups. **G** Representative micrographs of MBP-stained white matter in the striatum and hippocampus brain regions of the same animals together with (**H**) subcortical white matter loss measurements in the three different treatment groups. **I** Ipsilateral ventricular dilation measurements at P40 in naive, GMH + SAL and GMH + EX4 animals. Sample sizes were: Naive (*n* = 22), GMH + SAL (*n* = 33), GMH + EX4 (*n* = 33). Data presented as individual animals or mean ± SEM and analyzed using Kruskal–Wallis with Dunn’s post hoc test. *P < 0.05, **P < 0.01, ***P < 0.001 and ****P < 0.0001. Scale bar = 1 mm.

Histological assessment of brain injury 11 days after GMH revealed that GMH + EX4 animals had significantly less damage in gray matter (*P* = 0.0494; Fig. 5A, B) and subcortical white matter (*P* = 0.0068; Fig. 5C, D) compared to GMH + SAL animals. Further evaluation at 35 days after GMH (P40) showed that exendin-4 maintained reduced tissue loss both in gray matter (*P* < 0.0001; Fig. 5E, F) and white matter (*P* < 0.0001; Fig. 5G, H) when compared to saline-controls. Ipsilateral ventricular dilation at P40 demonstrated significant enlargement in GMH + SAL animals compared to naive controls (*P* = 0.0358). Exendin-4 significantly reduced ventricular size compared to GMH + SAL animals (*P* = 0.0310), with values comparable to naive animals (Fig. 5I).

### Exendin-4 improves early neurodevelopmental outcomes with long-term improvement of motor function

**Fig. 6:**
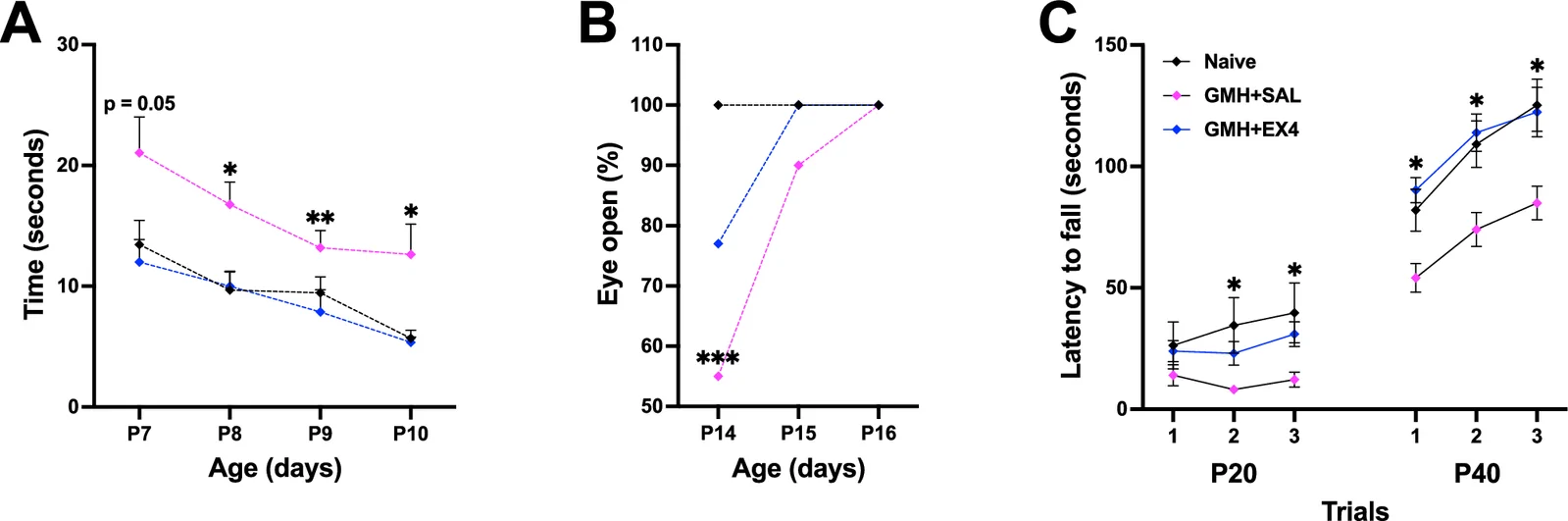
Exendin-4-treated animals show sustained functional improvement after P5 GMH injury. **A** Negative geotaxis early reflex locomotor test performed at P7-P10, **B** eye opening latency assessment was performed daily until P16 and **C** Rotarod motor function tests were performed at P20 and P40.

Negative geotaxis performed at P7-P10 revealed that GMH + SAL animals had a significant delay in sensorimotor competence when compared to naive controls at P8 (*P* = 0.011) and P10 (*P* = 0.03). Exendin-4 treatment significantly improved upright rotation when compared to GMH + SAL animals at all time-points: P7 (*P* = 0.0502), P8 (*P* = 0.039), P9 (*P* < 0.0001) and P10 (*P* < 0.0001; Fig. 6A). Subsequent neurodevelopmental assessments showed that ipsilateral eye opening occurred first in the naive animals, with both eyes open from P14. 74% of GMH + EX4 animals also had both eyes open at P14, reaching 100% at P15. However, GMH + SAL animals had significant delay in eye opening when compared to naive controls (*P* = 0.0007) with only 55% of their ipsilateral eye open at P14. By P15, 90% GMH + SAL animals had their eyes open and at P16, all animals had both eyes open (Fig. 6B). Overall, rotarod assessment showed that the GMH + SAL group had significant motor impairment with neuromuscular coordination deficits observed at both P20 (trial 2, *P* = 0.0104; trial 3, *P* = 0.042) and P40 (trial 1, *P* = 0.05; trial 2, *P* = 0.05; trial 3, *P* = 0.0185) time-points when compared to naive controls. Exendin-4 treatment resulted in an abolishment of this impairment where animals retained motor function similar to naive controls, with significant improvement when compared to GMH + SAL animals at both P20 (trial 1, *P* = 0.05; trial 3, *P* = 0.0405) and P40 (trial 1, *P* < 0.0001; trial 2, *P* = 0.0007; trial 3, *P* = 0.008) time-points (Fig. 6C). Cylinder rearing test revealed no effect in paw preference between the three groups (Supplementary Fig. 1).

Across behavioral, histological, and physiological/biochemical outcomes, we did not observe apparent sex-related differences in treatment response (data not shown).

## Discussion

The increased survival of infants born extremely preterm results in a higher number of cases suffering from PVH/IVH [7, 34]. GMH resulting in IVH/PVH represents a major clinical problem without established treatment options, and although many interventions have been explored experimentally, no therapy currently improves long-term neurological outcome in these infants [1]. Because GMH initiates secondary injury cascades involving regulated cell death and inflammation, targeting these mechanisms with exendin-4 represents a rational therapeutic approach.

In this study, we investigated whether our previously successful HI exendin-4 dosing regimen could attenuate GMH-mediated brain injury. Exendin-4 administered as a four-dose regimen starting immediately after GMH significantly reduced acute injury-associated markers, including activation of apoptotic pathways, inflammation, neutrophil infiltration, and MMP-9 expression. Importantly, treatment resulted in sustained protection of gray and white matter, reduced ventricular dilation [7], and improved neurological motor function at multiple developmental stages.

A key finding is that exendin-4 not only reduced early molecular injury markers but also maintained long-term anatomical and functional recovery. GMH is associated with neurodevelopmental delay and impaired motor function [7, 35, 36], particularly when hemorrhage extends into periventricular white matter [37]. In our model, exendin-4 normalized negative geotaxis righting and largely restored eye-opening timing [30, 38]. Rotarod performance [39–41] demonstrated recovery of motor coordination to levels comparable to naive animals. The functional improvement was accompanied by sustained preservation of gray and subcortical white matter. GLP-1R immunoreactivity was detected in both neurons and oligodendrocyte lineage cells, suggesting that exendin-4 may exert direct effects on these populations, thereby supporting both neuronal integrity and white matter preservation. Together, these findings show that exendin-4 provides sustained structural and functional protection in a perinatal GMH model.

GMH involves an initial hemorrhagic insult followed by secondary injury driven by blood product toxicity, oxidative stress [10], inflammation [30, 42–44], and activation of regulated cell death pathways [12, 45]. In the present study, measurement of hematoma size [33] confirmed that exendin-4 did not reduce the extent of the initial hemorrhage [46–48]. This was an important control to ensure that the GMH model remained intact and that the observed neuroprotection was not due to attenuation of the primary insult. Instead, the data indicate that exendin-4 acts downstream of the hemorrhagic event by modulating secondary injury pathways.

In terms of regulated cell death, exendin-4 significantly reduced caspase-3 and p53 expression, indicating attenuation of intrinsic apoptotic signaling [49, 50]. In parallel, AIF, a key mediator of caspase-independent cell death, was also reduced. Both caspase-dependent and AIF-mediated pathways are well-established contributors to neuronal loss in immature brain injury models [51, 52]. The concurrent suppression of these pathways suggests that exendin-4 may act upstream of executioner mechanisms; however, it remains unclear whether these effects reflect independent mechanisms or downstream consequences of a common upstream process. This is consistent with the reported anti-apoptotic effects of GLP-1R activation [53], which have been linked to modulation of mitochondrial dysfunction and pro-survival signaling pathways [54]. This is consistent with the receptor-dependence of exendin-4’s anti-apoptotic effects, which we have previously demonstrated using GLP-1R antagonism and in GLP-1R knockout models [26, 55]. The ability to attenuate both caspase-dependent and caspase-independent mechanisms may be particularly relevant in the immature brain [56, 57].

Inflammation and vascular dysfunction further amplify secondary injury after GMH. Hemorrhage results in breakdown of vessels surrounding the lesion, infiltration of neutrophils, and increased MMP-9 expression, leading to degradation of the endothelial basement membrane [43, 58]. Exendin-4 reduced IL-1β levels as early as 6 h post-GMH, indicating early suppression of pro-inflammatory signaling. This was accompanied at 24 h by reduced microglial activation, neutrophil infiltration and MMP-9 expression [59]. Since MMP-9 contributes to extracellular matrix degradation and blood–brain barrier disruption, its reduction suggests stabilization of the neurovascular unit. Consistent with this, qualitative immunofluorescence demonstrated preserved laminin and claudin colocalization in the peri-hemorrhagic region following exendin-4 treatment. MMP-9 is both induced by and amplifies inflammatory signaling [60], creating a cascading effect that promotes further tissue injury and cell death. These findings suggest that attenuation of inflammatory activation and MMP-9 expression by exendin-4 may be associated with reduction of the propagation of the secondary injury cascade and downstream activation of apoptotic pathways.

Exendin-4 treatment resulted in reduced weight gain, consistent with its known satiating effects [61], but this was transient and animals resumed parallel growth after treatment cessation. No hypothermia was observed. A transient increase in glucose occurred without changes in insulin, likely reflecting a species-specific response [62–64]. Mild ketonemia was observed, probably secondary to reduced caloric intake [65]. Clinically, neonatal caloric intake is tightly regulated [66], and exendin-4 is known to normalize glucose without causing hypoglycemia [67].

While these findings support a beneficial metabolic and neuroprotective profile of exendin-4 treatment, several limitations of the present study should also be considered. This study evaluated only a single dose level of exendin-4, and therefore whether lower or higher doses would produce comparable or superior neuroprotective effects remains unknown. Exendin-4 was administered within minutes of GMH induction, which does not reflect the clinical scenario in which the condition is typically identified hours after the hemorrhagic event. We have previously shown that exendin-4 retains neuroprotective efficacy when administration is delayed by 2 h in a neonatal hypoxia-ischemia model [25], but whether a comparable treatment window applies to GMH specifically remains to be tested. Although the present study did not include in vivo GLP-1R antagonism or knockout experiments specific to the GMH model, receptor-dependent mechanisms of exendin-4-mediated neuroprotection have been established in both in vitro and in vivo settings in our previous hypoxia-ischemia model work. The GLP-1R antagonist exendin-9 blocked exendin-4-mediated normalization of HIF-1α, ATF3 and CREB expression and significantly attenuated, although did not fully abolish, the associated reduction in caspase-3, -8 and -9 activity and improvement in neuronal survival following oxygen-glucose deprivation in vitro, and also reversed exendin-4-mediated neuroprotection in vivo in a neonatal hypoxia-ischemia model [26]. Furthermore, exendin-4-mediated neuroprotection in neuronal cultures is abolished in cells derived from GLP-1R knockout mice [55]. In addition, functional assessment in this study focused on sensorimotor and motor outcomes; cognitive and behavioral domains relevant to long-term GMH morbidity, including learning and memory [68, 69], were not evaluated and represent an important direction for future work. Large animal validation is also essential to confirm translational relevance; we have recently shown that exendin-4 monotherapy is neuroprotective in a rabbit model of hypoxia-ischemia [27], and extending this work to a large animal model of GMH specifically would further support clinical translation.

In summary, exendin-4 attenuates converging secondary injury mechanisms following GMH, including caspase-dependent and AIF-mediated regulated cell death, inflammation, and neurovascular disruption. This early modulation translates into sustained structural preservation and long-term functional recovery. These findings identify GLP-1R activation as a promising strategy to target regulated cell death and inflammatory amplification in neonatal hemorrhagic brain injury. Nevertheless, validation in large animal models that more closely replicate the developmental and vascular characteristics of the preterm human brain is required before clinical translation.

## Supplementary information

Supplementary Figure 1

aj-checklist

Supplementary figure 1

## Data Availability

The data sets will be available upon reasonable request.
