# Supplementary material for: Exendin-4 improves neurodevelopmental outcome after neonatal germinal matrix hemorrhage
Source: Cell Death Dis. 2026 Aug 19;17(1):743. doi: 10.1038/s41419-026-09189-9 (PMC13490559; doi:10.1038/s41419-026-09189-9)
Supplement: Supplementary file 1 [file 41419_2026_9189_MOESM1_ESM.pdf]

A

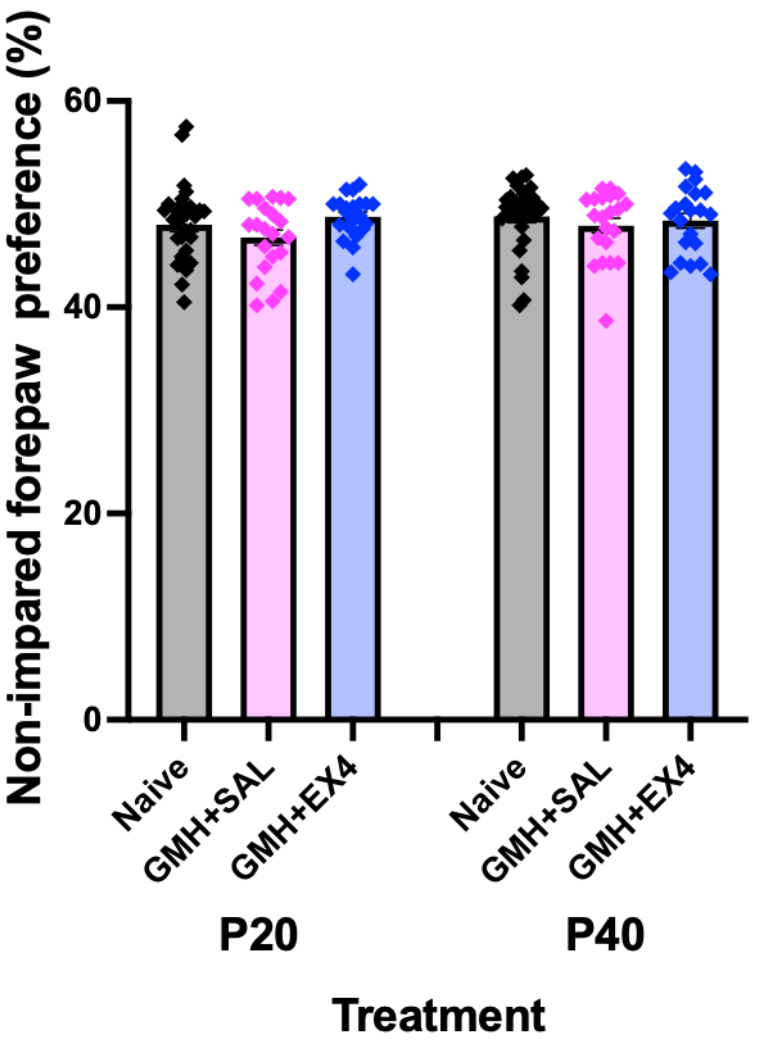
Non-impared forepaw preference (%)
60
40
20
0
Naive
GMH+SAL
GMH+EX4
Naive
GMH+SAL
GMH+EX4
P20
P40
Treatment
