# Supplementary material for: Exendin-4 improves neurodevelopmental outcome after neonatal germinal matrix hemorrhage
Source: Cell Death Dis. 2026 Aug 19;17(1):743. doi: 10.1038/s41419-026-09189-9 (PMC13490559; doi:10.1038/s41419-026-09189-9)
Supplement: Supplementary file 2 [file 41419_2026_9189_MOESM2_ESM.pdf]

Corresponding Author Name: Eridan Rocha-F
Manuscript Number: CDDIS-26-1923

SPRINGER NATURE

Reporting Summary

Springer Nature wishes to improve the reproducibility of the work that we publish. This checklist is used to ensure good reporting standards and to improve the reproducibility. Please respond completely to all questions relevant to your manuscript. For more information, please read the journal's Guide to Authors.

☐ **Check here to confirm that the following information is available in the Material & Methods section:**

- **The exact sample size (*n*)** for each experimental group/condition, given as a number, not a range
- **A description of the sample collection** allowing the reader to understand whether the samples represent technical or biological replicates (including how many animals, litters, culture, etc.)
- **A statement of how many times the experiment shown was replicated in the laboratory**
- **Definitions of statistical methods and measures**: For small sample sizes (n<5) descriptive statistics are not appropriate, instead plot individual data points
  - Very common tests, such as *t*-test, simple χ^2 tests, Wilcoxon and Mann-Whitney tests, can be unambiguously identified by name only, but more complex techniques should be described in the methods section
  - Are tests one-sided or two-sided?
  - Are there adjustments for multiple comparisons?
  - **Statistical test results**, e.g., ***P* values**
  - Definition of **'center values'** as **median or mean**;
  - Definition of **error bars as s.d. or s.e.m. or c.i.**

Please ensure that the answers to the following questions are reported in the manuscript itself. We encourage you to include a specific subsection in the methods section for statistics, reagents and animal models. Below, provide the page number or section and paragraph number.

Statistics and general methods

	Reported in section/paragraph or page #
1. How was the sample size chosen to ensure adequate power to detect a pre-specified effect size? (Give section/paragraph or page #)	No formal a priori sample size calculation was performed. Group sizes were based on previous experience with the P5 GMH model and prior studies using exendin-4, with the aim of detecting biologically relevant effects while minimizing
For animal studies, include a statement about sample size estimate even if no statistical methods were used.	No formal a priori sample size calculation was performed. Group sizes were determined prior to the start of the experiments based on previous experience with the P5
2. Describe inclusion/exclusion criteria if samples or animals were excluded from the analysis. Were the criteria pre-established? (Give section/paragraph or page #)	No animals or data points were excluded from the analyses. This is not explicitly stated in the manuscript.
3. If a method of randomization was used to determine how samples/animals were allocated to experimental groups and processed, describe it. (Give section/paragraph or page #)	Animals were weighed and sexed prior to allocation and assigned to experimental groups in a randomized and balanced manner within each litter. Methods, page 6.
For animal studies, include a statement about randomization even if no randomization was used.	Animals were weighed and sexed prior to allocation and assigned to experimental groups in a randomized and balanced manner within each litter.

4. If the investigator was blinded to the group allocation during the experiment and/or when assessing the outcome, state the extent of blinding. (Give section/paragraph or page #)	Outcome assessments were performed by an investigator blinded to group allocation. Methods, page 10.
For animal studies, include a statement about blinding even if no blinding was done.	Outcome assessments were performed by an investigator blinded to group allocation.
5. For every figure, are statistical tests justified as appropriate?	Yes. Statistical tests were selected based on data distribution and experimental design, as described in the Statistics section. The specific statistical test used for each
Do the data meet the assumptions of the tests (e.g., normal distribution)?	Data were tested for Gaussian distribution using the D’Agostino & Pearson test, and appropriate parametric or non-parametric tests were applied accordingly.
Is there an estimate of variation within each group of data?	Yes. Data are presented as mean ± SEM, as described in the Statistics section and indicated in the figures.
Is the variance similar between the groups that are being statistically compared? (Give section/paragraph or page #)	Statistical analyses were performed based on data distribution, and appropriate tests were selected accordingly. Methods, page 11; figure legends.

Reagents

	Reported in section/paragraph or page #
6. Report the source of antibodies (vendor and catalog number)	All antibodies used in the study are reported with vendor, catalog number, and dilution in the Methods section. Methods, pages 8-10.
7. Identify the source of cell lines and report if they were recently authenticated (e.g., by STR profiling) and tested for mycoplasma contamination	No cell lines were used in this study.

Animal Models

	Reported in section/paragraph or page #
8. Report species, strain, sex and age of animals	Wistar rats of both sexes were used. Germinal matrix hemorrhage was induced at postnatal day 5 (P5).
9. For experiments involving live vertebrates, include a statement of compliance with ethical regulations and identify the committee(s) approving the experiments.	All animal experiments were approved by the Swedish Board of Agriculture and the Gothenburg Animal Ethics Committee (825-2017, 3033-2020 and 4031/22) and were conducted in accordance with ARRIVE guidelines and relevant institutional regulations.

10. We recommend consulting the ARRIVE guidelines (*PLoS Biol.* **8**(6), e1000412,2010) to ensure that other relevant aspects of animal studies are adequately reported.

Human subjects

Reported in section/paragraph or page #

11. Identify the committee(s) approving the study protocol.	Not applicable. This study involved only animal experiments and no human subjects.
12. Include a statement confirming that informed consent was obtained from all subjects.	Not applicable.
13. For publication of patient photos, include a statement confirming that consent to publish was obtained.	Not applicable
14. Report the clinical trial registration number (at ClinicalTrials.gov or equivalent).	Not applicable

15. For phase II and III randomized controlled trials, please refer to the CONSORT statement and submit the CONSORT checklist with your submission.

16. For tumor marker prognostic studies, we recommend that you follow the REMARK reporting guidelines.

Data deposition

Reported in section/paragraph or page #

17. Provide accession codes for deposited data. Data deposition in a public repository is mandatory for:<br>a. Protein, DNA and RNA sequences<br>b. Macromolecular structures<br>c. Crystallographic data for small molecules<br>d. Microarray data	Not applicable. No datasets requiring deposition in a public repository were generated in this study.

Deposition is strongly recommended for many other datasets for which structured public repositories exist; more details on our data policy are available in the Guide to Authors. We encourage the provision of other source data in supplementary information or in unstructured repositories such as Figshare and Dryad. We encourage publication of Data Descriptors (see Scientific Data) to maximize data reuse.

18. If computer code was used to generate results that are central to the paper's conclusions, include a statement in the Methods section under "**Code availability**" to indicate whether and how the code can be accessed. Include version information as necessary and any restrictions on availability.	Not applicable. No custom computer code was used to generate results central to the conclusions of this study.
