# Supplementary material for: Exendin-4 improves neurodevelopmental outcome after neonatal germinal matrix hemorrhage
Source: Cell Death Dis. 2026 Aug 19;17(1):743. doi: 10.1038/s41419-026-09189-9 (PMC13490559; doi:10.1038/s41419-026-09189-9)
Supplement: Supplementary file 3 [file 41419_2026_9189_MOESM3_ESM.docx]

**Supplementary figure 1. (A)** Cylinder test assessing forelimb preference was performed at P20 and P40.
